# Role of Lipopolysaccharide, Derived from Various Bacterial Species, in Pulpitis—A Systematic Review

**DOI:** 10.3390/biom12010138

**Published:** 2022-01-15

**Authors:** Aniela Brodzikowska, Monika Ciechanowska, Michał Kopka, Albert Stachura, Paweł K. Włodarski

**Affiliations:** 1 Department of Conservative Dentistry, Medical University of Warsaw, 02-091 Warszawa, Poland; aniela.brodzikowska@wum.edu.pl; 2 Department of Methodology, Medical University of Warsaw, Żwirki i Wigury 61, 02-097 Warszawa, Poland; nika.ciechanowska@gmail.com (M.C.); m_kopka@wp.pl (M.K.); albert.stachura@wum.edu.pl (A.S.); 3 Doctoral School, Medical University of Warsaw, 02-091 Warszawa, Poland

**Keywords:** LPS, pulpitis, inflammation, pulp cells

## Abstract

Lipopolysaccharide (LPS) is widely used for induction of inflammation in various human tissues, including dental pulp. The purpose of this study was to summarize current medical literature focusing on (1) cell types used by researchers to simulate dental pulp inflammation, (2) LPS variants utilized in experimental settings and how these choices affect the findings. Our study was conducted in accordance with the Preferred Reporting Items for Systematic Reviews and Meta-Analyses (PRISMA). We searched for studies reporting outcomes of lipopolysaccharide application on dental pulp cells in vitro using electronic databases: MEDLINE, Web of Science and Scopus. Having gathered data from 115 papers, we aimed to present all known effects LPS has on different cell types present in dental pulp. We focused on specific receptors and particles that are involved in molecular pathways. Our review provides an essential foundation for further research using in vitro models of pulpitis.

## 1. Introduction

Pulpitis is an inflammation of the dental pulp—a painful condition mostly caused by Gram-negative bacteria. Lipopolysaccharides, which are components of the Gram-negative bacterial outer membrane, take part in inducing inflammation in the dental pulp. Understanding mechanisms underlying this pathology may help find novel specific treatments.

### 1.1. Cells in Dental Pulp

Dental pulp is a loose connective tissue of ectomesenchymal origin, enclosed by three mineralized materials: dentin, enamel, and cementum [1]. It consists various cells: fibroblasts, odontoblasts, macrophages, mast cells and plasma cells [2]. The odontoblast layer lying on the periphery, at the interface of dentin-pulp complex, is responsible mainly for dentin development [2]. Channels created by odontoblasts extend through dentin and provide nutrient to avascular dentin [2]. Beneath the odontoblast layer lies the cell-poor zone (zone of Weil) [2] and underneath—a cell-rich zone, filled with fibroblasts and undifferentiated mesenchymal cells [2]. At the core, the central pulp contains numerous fibroblasts, vessels and autonomic and sensory nerves, which extend to peripheral layers [2].

### 1.2. Pulpitis

Pulp inflammation is a dynamic and complex process involving neural, vascular and immunological reactions. Microorganisms, dentinal tubules of the teeth, chemical or mechanical irritation, as well as trauma may induce this state [1].

Bacterial infection is the most frequent cause of pulp diseases. Pulp inflammation is primarily the result of cooperation between anaerobic bacteria (such as *Porphyromonas gingivalis*, *Prevotella intermedia*, *Fusobacterium nucleatum *and* Treponema* *denticola*) [3] and other Gram-negative bacteria [4]. Thus far, bacteria species have not been correlated with a specific clinical symptomatology [3]. The pulpitis can be reversible when it subsides after treatment, or irreversible when healing is not possible [2].

### 1.3. Lipopolysaccharide

Lipopolysaccharides are components of the outer membrane of Gram-negative bacteria, composed of a hydrophobic domain—endotoxin—lipid A, hydrophilic O-antigen and core polysaccharide [5]. The most bioactive LPS element is lipid A [5]. LPS by binding to cell surface receptors, such as Toll-Like Receptor 4 (TLR4)/Cluster of Differentiation 14 (CD14)/Myeloid Differentiation factor 2 (MD2), induces the production and secretion of proinflammatory cytokines, nitric oxide and eicosanoids [5]. LPS is used in research to induce inflammation in human dental pulp cells in vitro.

Here we review types of LPS used in research and the mechanisms underlying their effect on different cell types—especially human dental pulp cells (hDPCs), human dental pulp stem cells (hDSPCs), fibroblasts and odontoblasts.

## 2. Materials and Methods

This study was conducted in accordance with the Preferred Reporting Items for Systematic Reviews and Meta-Analyses (PRISMA) guidelines, using a previously designed protocol (Supplementary File S1). We searched for studies reporting outcomes of lipopolysaccharide influence on dental pulp cells in vitro. All articles written in English, except for reviews, letters, and editorials, were included.

**Figure 1:**
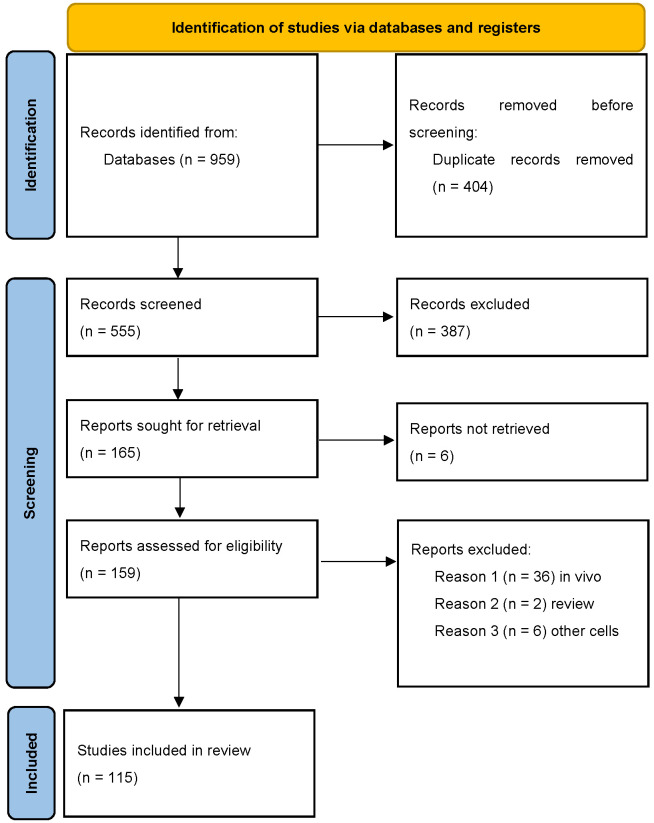
PRISMA flowchart describing data collection for this study.

We searched using electronic databases: MEDLINE, Web of Science and Scopus. To identify all relevant articles, we used prespecified search engines for each database (Figure 1, Supplementary File S1). Additionally, we screened references of selected articles to find papers not identified in the primary search. The systematic search of the literature was performed by M.C. and M.K. independently in October 2021.

### 2.1. Study Selection

Each relevant publication was categorized, based on cellular cultures, interventions used and outcomes. Articles were included based on predefined selection criteria: appropriate design (LPS use in induction of inflammation in vitro), reporting of the outcomes and a defined cellular culture. Exclusion criteria were in vivo studies, reviews, letters and editorials, inadequate design, and a substantial lack of methodology.

Study eligibility was assessed by screening titles and if necessary, abstracts. Later, full texts were assessed for inclusion and exclusion criteria. All disagreements were resolved by a consensus between the two reviewers (M.C. and M.K.).

### 2.2. Data Extraction and Analyses

The following information was extracted from each study by M.C and M.K: LPS type, cellular culture type and outcomes. Strengths and weaknesses of selected studies were appraised in the discussion.

We did not perform quantitative statistical analysis of selected studies because of methodological and clinical heterogeneity. A systematic review of the outcomes was undertaken instead.

## 3. Results

### 3.1. Escherichia coli LPS

#### 3.1.1. hDPCs

*E coli* LPS was used in the most investigated studies. It induces cytokine expression: Interleukin 1β (IL-1β), IL-6, IL-8 and tumor necrosis factor α (TNF-α) and extracellular matrix-associated proteins: matrix metalloproteinases (MMPs) [6], vascular cell adhesion molecule 1 (VCAM-1) and Intercellular Adhesion Molecule 1 (ICAM-1). The above-mentioned changes are mediated by TLR4 receptor activation and nuclear factor kappa-light-chain-enhancer of activated B cells (NF-κB) pathway.

Some studies found alternations in transcriptional factors upon LPS stimulation. One of upregulated proteins is ten-eleven translocation 2 (TET2) DNA methylcytosine dioxygenase [7], responsible for hydroxymethylation of e.g., Myeloid differentiation primary response 88 (MyD88) gene and promoting its expression. LPS stimulation also upregulates octamer-binding transcription factor 4-B1 (OCT4B1) leading to overexpression of OCT4B [8]. Both particles protect hDPCs from apoptosis [9]. Silencing OCT4B1 changes the expression of 38 microRNAs [8]. Upon inflammation onset, DNA methyltransferase 1 (DNMT1) expression diminished, leading to changes in methylation of IL-6 and TNF-receptor-associated factor-6 (TRAF6) promoters, thus activating NF-κB and mitogen-activated protein kinase (MAPK) pathways [10]. A similar pro-inflammatory effect is observed when using 5-Aza-2′-deoxycytidine (5-Aza-CdR), which inhibits methyltransferases [11].

LPS may regulate noncoding RNA particles. The maternally expressed gene 3 (MEG3) particle belonging to long non-coding RNA (lncRNA) group is induced in inflamed dental pulp and LPS-treated cells. Knocking down this lncRNA stopped proinflammatory proteins expression, possibly by disturbing the p38/MAPK signaling pathway [12]. MEG3 depletion promoted odontogenic differentiation of hDPCs via Wnt/βcatenin pathway [12]. Another lncRNA group member, upregulated by LPS, is NUTM2A antisense RNA 1 (NUTM2A-AS1) [13]. Silencing its target—let-7c-5p—promotes High-Mobility Group Box 1 (HMGB1) expression [13]. This protein decreases viability and proliferation of hDPCs [14] but promotes cellular migration [13]. LPS-induced inflammation is also regulated by microRNAs. Overexpression of microRNA-21-5p mitigates activation of NF-κB and cytokine expression—an effect likely mediated by targeting UTR region of TRAF6, which is a part of TLR4 transduction pathway [15].

LPS induces reactive oxygen species (ROS) production [16] and downregulates ROS-scavenging protein peroxisome proliferator-activated receptor γ (PPARγ). Subsequent activation of NF-κB and ERK1/2 increases expression of VCAM-1, ICAM-1 and MMPs [17,18]. Rosiglitazone (PPARγ agonist) attenuates proinflammatory state by reducing cellular NO activity and ROS formation [17]. LPS also upregulates astrocyte elevated gene-1 (AEG-1) and activates nuclear translocation of p65 [19]. Hypoxia-inducible factor 1α (HIF-1α) plays a double role in inducing inflammation. Its overexpression leads to increased IL-1β and TNF-α production [20]. At the same time, HIF-1α downregulates IL-6 [21]. This phenomenon occurs because HIF-1α activates the suppressor of cytokine signaling 3 (SOCS3), which leads to downregulating CCAAT enhancer-binding protein beta (CEBPb). Since CEBPb is an inducer of IL-6, hence the net result is IL-6 downregulation [21].

*E. coli* LPS-induced pulpal cells are also used as a model for searching potential anti-inflammatory compounds. One such compound is involved in apoptosis regulation. Sirtuin 6 (SIRT6) belonging to NAD+-dependent deacetylases, regulates Ku70 acetylation [22]. Overexpression of SIRT6 leads to deacetylation of Ku70, interrupting mobilization of an apoptosis inductor—Bax protein [22]. The second compound group is associated with autophagy. LPS decreases levels of autophagy mediators—autophagy related 5 protein (ATG-5), Beclin-I, microtubule-associated protein 1A/1B light chain 3 (LC3-I/II), mitochondrial biogenesis molecules heme oxygenase-1 (HO-1) and peroxisome proliferator-activated receptor gamma coactivator 1-alpha (PGC-1α) [23]. Schisandrin C is a natural product extracted from Schisandra chinensis, commonly used in Chinese medicine. This compound restores the expression of the above-mentioned proteins [23]. Schisandrin C activates autophagy, leading to osteoblastic differentiation of hDPCs, mediated by upregulated dentin matrix acidic phosphoprotein 1 (DMP-1), dentin sialophosphoprotein (DSPP), Bone morphogenetic protein 2 (BMP-2) and BMP-7 [23]. Davallialactone [24] (hispidin analogue), produced by the mushroom Inonotus xeranticus, is a potent antioxidant [25]. Davalillialactone decreases ROS activity in LPS-stimulated cells [24]. It also inhibits nuclear translocation of NF-κB and ERK1/2 activity and mitigates expression of proinflammatory particles i.e., MMP-2, MMP-9, VCAM-1, ICAM-1, inducible nitric oxide synthases (iNOS) and cyclooxygenase (COX2) [24]. A similar mechanism of action is presented by teneligliptin, dipeptidyl peptidase-4 inhibitor [26]. It decreases production of 4-Hydroxynonenal (4-HNE) from lipid peroxidation, which can represent ROS activity. Teneligliptin also reduces expression of TLR4 [26]. Another study showed that hDPCs treated with NAC (N-acetylcysteine), combined with Biodentine or Mineral trioxide aggregate (MTA) (cement compounds used in pulpitis treatment) [27], showed better viability. The group with MTA and NAC treatment also promoted mineralization [27].

A significant group of anti-inflammatory compounds is related to inhibition of NF-κB pathway. Fluocinolone acetonide (FA) is a steroidal anti-inflammatory drug [28]. In LPS-stimulated cells it reduces expression of IL-1β, IL-6 and IL-8 and up-regulates odontoblastic markers—alkaline phosphatase (ALP), Runt-related transcription factor 2 (RUNX2) and DSPP [28]. FA also increases PPARγ mRNA expression by inhibiting the expression of phosphorylated-NF-κB P65 and triggering activator protein-1 (AP-1) [28]. Another study by Wang et al. showed that betamethasone (steroid), added to LPS-treated cells, reduces expression of IL-1β, IL-6 and TNF-α and promotes mineralization markers—ALP, DSPP and osteocalcin [29]. Simvastatin, a lipid-lowering medication, significantly reduces LPS-stimulated production of IL-1β, IL-6, VCAM-1 and ICAM-1 [30]. It also decreases expression of p65, its phosphorylation and nuclear translocation. Simvastatin mitigates phosphorylation of I-κB [30]—an effect also observed for *P. gingivalis* LPS.

Nel-like molecule type 1 (Nell-1) is a compound used as an osteoinductive factor. Human recombinant Nell-1 attenuates p38 and ERK MAPK pathways and decreases expression of IL-6 and IL-8 in LPS-stimulated cells [31].

Bromelain is a natural product, containing various bioactive substances e.g., thiol endopeptidases, phosphatase, glucosidase, peroxidase, cellulase, and several protease inhibitors and is obtained from pineapple (*Ananas comosus*) stems and fruits [32]. It decreases IL-1β, IL-6, IL-8, ICAM-1 and VCAM-1 by reducing phosphorylation of p65, ERK and p38. It also promotes ALP activity and formation of mineralized nodules [33]. A similar effect is exerted by terrein [34]. This *Penicillum* sp. bioactive metabolite reduces production of VCAM-1 and ICAM-1 by blocking the activation of Akt and suppressing NF-κB [34].

Catechins—polyphenols derived from green tea have an anti-inflammatory potential [35]. Epigallocatechin-3-gallate (EGCG) and epicatechin gallate (ECG) reduce production of IL-6, IL-8, VCAM-1, ICAM-1, VEGF and COX-2, induced by LPS [36,37].

Acemannan, a polysaccharide from Aloe vera induces proliferation, differentiation, growth factor and ECM synthesis and formation of mineralized bridge [38].

Huang et al. showed that hDPCs treated with LPS and co-stimulated with odontogenic induction medium had increase ALP activity, DSPP DMP-1 via NF-κB and no change in c-Jun expression [39].

LPS stimulates production of IL-1β, IL-6. After 14 days, cultured cells presented markers of dentinogenesis. Wnt5a, Runx2 and ALP proteins were upregulated, and ALP activity was increased on the 21st day. Wnt5 activation was confirmed by using inhibitor—Box5, which attenuated Runx2 and ALP levels [40].

#### 3.1.2. Fibroblasts

*E. coli* LPS was used in 7 out of 13 experiments performed on human pulp fibroblasts. Bacteria serotypes were specified in 5 articles—*E. coli* 055:B5 in 2, L-2880 in 1, 0111:B4 in 2.

*E. coli* LPS stimulates the production of pro-inflammatory factors: CCL2, IL-8 and IL-6 [41]. Less intense upregulation occurs for IL-4, GCFS, GM-CSF and CCL5, as well as for IL-1β, IL-10, IL12p70, IL-17A, TNF-α, INF-λ [41] and TNFβ [42]. The maturation and release of IL-1β is stimulated by a combination of LPS and ATP. This effect is mediated by stimulating the pyrogenic P2X7 ATP-gated ion channel and subsequent activation of the NLRP3/caspase-1 pathway [42,43]. The process involves two steps: (1) activation of TLR4/MyD88/NF-ϰB pathway by LPS resulting in up-regulation of NLRP3 and expression of pro-IL-1β genes, and (2) ATP-stimulated ROS production is the second signal for NLRP3 activation [44]. *E. coli* LPS stimulates COX-2, but not COX-1 [45].

MicroRNAs take part in the posttranscriptional regulation of gene expression. LPS stimulation increases expression of miR-146a (the miRNA precursor) in gingiva fibroblasts, periodontal ligament fibroblasts and dental pulp cells, upregulating KBTBD7 (gene encoding a transcriptional activator) and downregulating miR-21 and miR-155 [46,47].

Considering inflammation, methyl mercaptan (CH3SH) acts synergically with LPS, stimulating IL-6 production [42]. Exogenous melatonin causes an opposite effect via suppression of COX-2 and IL-1β [45]. Berberine reduces IL-1β, IL-6 and TNF-α expressions, proliferation and miR-21 expression, inhibiting inflammatory response [47,48].

#### 3.1.3. Odontoblasts

*E. coli* LPS was used in four out of eight experiments with odontoblast-like cells. Serotypes were specified in three articles—*E. coli* 055:B5, *E. coli* k-235 strain LPS and *E. coli* L-2880.

Two receptors—TLR4 and CD14—bind LPS on the surface of odontoblasts [49]. LPS stimulation results in IL-8 mRNA and protein expression [50]. Binding to TLR4 upregulates VEGF (regulator of angiogenesis) expression in odontoblasts [49]. It can be associated either with regeneration of the pulp-dentinal complex or with necrosis caused by vessel collapse [49]. Odontoblasts are involved in alleviating inflammation in surrounding tissues. In response to LPS, they produce secretory leukocyte protease inhibitor (SLPI), which inhibits the activation of NF-kB and antagonizes LPS response [51]. Both mechanical injury and LPS stimulation are associated with similar Notch signaling pathway activation, which prompts differentiation of precursor cells to odontoblast-like cells [52]. This mechanism protects against injury, prevents apoptosis, enhances cell survival and maturation [52]. LPS induces inflammatory cytokines and chemokines in odontoblast-like cells, macrophages and osteoclasts. Propolis decreases production of IL-1a, MIP-1a, IL-12(p70) and IL-15 in odontoblast-like cells; MIP-1α, G-CSF, TNF-α and IL-6 in macrophages and MIP-1α production in osteoclasts [53].

A study of Liao et al. investigated the role of sclerostin in LPS induction. Upon LPS stimulation, production of sclerostin increased, impairing odontoblastic differentiation. Sclerostin activates NF-κB pathway, upregulating expression of IL-6, IL-8, IL-1β, ICAM-1, VCAM-1, CXCR4, MCP-1, VEGF, VEGFR-1 and PlGF [54].

#### 3.1.4. DPSCs

*E. coli* LPS was used in 12 out of 25 experiments performed using dental pulp stem cells (DPSCs). Serotypes were specified in 6 articles—*E. coli* 055:B5 in two, L-2880 in one, 0111:B4 in three articles.

Bindal et al. (2018) concluded that treating hDPSCs cells with 1 μg/mL *E. coli* LPS for 24 h is the most appropriate approach to inducing an inflammatory microenvironment, based on IL-6, IL-8, tPA and TAC1 levels [55]. LPS alone did not promote production of IL-6 in the initial phase (after 48 h), but after 7 days, rising LPS concentrations significantly increased secretion of Il-6 [56]. IL-1α level was increasing proportionally to LPS concentrations, suggesting a progressive onset of inflammation [55]. Uncontrolled overexpression of IL-1β may be the primary force driving the inflammation’s severity and causing pulp necrosis [57]. Interestingly, the level of TNFα was significantly increased despite a lack of genetic expression. This phenomenon could be explained by inflammation initiation by *E. coli* LPS via a dual pathway—TLR4 and TLR2 [55].

Innate immune response, especially mediated by macrophages, is modulated in DPSCs through the TNF-α/IDO axis [57]. LPS-induced TLR4 activation leads to NF-κB p65 translocation into the nucleus, with subsequent expression of iNOS, COX-2 and inflammatory mediators, such as TNF-α. The expression of the NF-κB subunit p65 is partially blocked by DPSCs and reduces TNF-α production by macrophages. IDO expression levels in DPSCs are increased after LPS or TNF-α stimulation in a time-dependent manner [57]. These mechanisms may be associated with an anti-inflammatory effect of DPSCs.

##### Differentiation

Wnt5a induces mesenchymal stem cells osteogenesis and suppresses adipogenesis; plays a role in tooth development and odontoblast differentiation [58]. It also upregulates inflammatory cytokines e.g., IL-6, IL-8 and IL-1b activating the non-canonical pathway [58]. LPS can enhance Wnt5a expression in a time and dose-dependent manner in hDPSCs—an effect mediated by TLR4/MyD88, PI3K/AKT and NF-kB signaling pathways [58]. LPS promotes mRNA expression of genes related to mineralization, such as OCN, DSPP, ALP and BSP in DPSCs obtained from rats and this effect declines with age [59]. LPS inhibits osteogenic differentiation of DPSCs via HMGA2/PI3K/Akt pathway [60].

He et al. (2014) [61] demonstrated that odontogenic differentiation was associated with TLR4 activation by LPS in hDPSCs. They also proved that activation of p38 mitogen-activated protein kinase (MAPK) and extracellular signal-regulated kinase (ERK), but not NF-κB, signaling pathways are involved in LPS-mediated differentiation of hDPSCs [61]. LPS alone promotes the migration of DPSCs, however 4-Methylumbelliferone (4-mu) can further accelerate the migration and odontogenetic differentiation by downregulating the expression of inflammatory cytokines [62].

Tissue-committed stem cells (TCSCs) migrate to injury sites and differentiate there, prompted by stromal cell–derived factor -1a (SDF-1a)/CXC chemokine receptor 4 (CXCR4) axis [63]. Secretion of SDF-1 may be enhanced (directly by LPS) or suppressed (indirectly by inflammatory cytokines) [63]. Inducing cells with LPS increased the production of CXCR4 [63]. Both SDF-1 and CXCR4 are regulated by hypoxia [63].

##### Senescence

LPS-treated DPSCs age faster—they have a flat shape, increased size and disordered F-actin, surrounding the nucleus [64]. Upon LPS stimulation, levels of senescence markers, e.g., β-galactosidase (SA-b-gal) rise in dose-dependent manner [65]—a finding associated with TLR4/MyD88-NF-κB-p53/p21 signaling pathways [65]. p65 knockdown weakens the NF-κB signal and reverses the senescence of DPSCs by enhancing the proliferation [65].

Another mechanism involved in cellular senescence is DNA damage. Excess ROS cause oxidative DNA damage (increased γ-H2A.X expression—a sensitive marker for DNA damage [64,66]), also directly activating p16INK4A expression, stopping the cell cycle [64]. LPS induces double strand breaks and subsequent DNA damage [66]. Two main classic DNA repair pathways in eukaryotic cells are homologous recombination (HR) and non-homologous end joining (NHEJ). The mRNA and protein expression levels of Ku70 and Xrcc4 involved in NHEJ, and Rad51 in HR, are increased in LPS-treated DPSCs [66]. Inhibiting Ku70 promotes the p53 pathway apoptosis [66].

LPS has a diverse effect on cell viability, defined as active cell metabolism. Some authors suggest it reduces cell viability [55], while others postulate it has no such effect [56]. Nonetheless, LPS treatment decreases cell survival by increasing apoptosis and necrosis rates [56]. LPS promotes proliferation of DPSCs by increasing TLR4 expression and through HMGA2/PI3K/Akt pathway [59,60].

Some agents act antagonistically to *E. coli* LPS in hDPSCs. 4-Methylumbelliferone (4-mu) downregulates the expression of inflammatory cytokines (decreased CD44 expression, preventing LMW HA-TLR4-CD44 complex formation) and facilitates cell differentiation [62]. Epigallocatechin gallate (EGCG) has an anti-inflammatory effect, not affecting cell proliferation or differentiation [67]. Betamethasone blocks NF-κB activation, alleviating inflammation and has osteo-/odonto-inductive effects on DPSCs [68]. Let-7c-5p could suppress the inflammatory phenomena and restore the osteogenic differentiation potential of inflamed DPSC via HMGA2/PI3K/Akt pathway [60].

As mentioned before, simvastatin presents anti-inflammatory features in hDPCs. It alleviates inflammation by downregulating TNF-α, IL-1β and MMP-9 [69]. It also increases the production of PPARγ, which is inhibited by LPS. Constructs of nanofibrous poly-(L-lactic acid) scaffolds with simvastatin trigger expression of proteins characteristic for odontoblastic differentiation [69]. This effect is mediated via phosphorylation of ERK1/2 and Smad1 [69].

### 3.2. Porphyromonas gingivalis LPS

#### 3.2.1. hDPCs

*P. gingivalis* LPS was used in seven studies.

LPS may influence global gene expression not only via TLRs activation, but also by changing the DNA methylation profile. *P. gingivalis* LPS downregulates mRNA level of DNA methyltransferase 3B (DNMT3B) and both mRNA and protein levels of DNA methyltransferase 1 (DNMT1) [70]. DNMTs inhibitor—5-Aza-CdR was used in these studies. Deregulation of above-mentioned particles increases levels of proinflammatory mediators and changes microRNAs expression [70]. The most significant change is the upregulation of miR-146a-5p, associated with pulp inflammation [71]. DNMT1 knockdown results in a similar methylation pattern of MIR146A promoter as obtained using 5-Aza-CdR [70]. LPS also induces the expression of methyltransferase-like 3 (METTL3) and extends the level of methylation at the N6 position of adenosine(m6A) [72]. Silencing METTL3 decreases the level of proinflammatory cytokines and inhibits NF-κB pathway. m6A and METTL3 regulate splicing of mRNA. Depletion of METLL3 causes production of MyD88 splice variant, which interrupts the TLR pathway [72].

IFNβ1 plays an essential role in inflamed cells. LPS stimulation increases NOD-like receptor family pyrin domain containing 6 (NLRP6), caspase 1 (Casp1) and Caps 4 expression, preceded by up-regulation of IFN-B and activation of IFNAR1 receptor [73]. Blocking this receptor cuts the signalization pathway. Similarly to previously-mentioned caspases, Casp3 is upregulated upon LPS stimulus [74], leading to mitigating SIRT6 expression. SIRT6 regulates deacetylation of Ku70, protecting against apoptosis [74]. Vascular endothelial growth factor (VEGF) and SIRT1 are upregulated after LPS stimulation [75]. It leads to activating angiogenesis, enhanced production of MMP-2 and MMP-9, induced migration, and formation of tube-like structures [75]. These processes are promoted by MAP kinases (p38, ERK, JNK), NF-κB and PIK3 pathways [75]. Muramyle dipeptide, which activates nucleotide binding oligomerization domain (NOD) 2, acts synergically with TLR2 and TLR4, enhancing expression of inflammatory particles e.g., human Beta-defensin 2 or COX2, prostaglandin E2 (PGE2), TNF-α, IL-6 and IL-8 [76].

An anti-inflammatory potential of several compounds were verified using *P.gingivalis* LPS. GV1001 oligopeptide is a molecule similar to a part of human reverse transcriptase subunit of telomerase (hTERT), which is a potential target for cancer vaccines [77]. GV1001 decreases LPS-induced phosphorylation of ERK and p38 MAPK, inhibiting TNF-α and IL-6 production [78]. Previously mentioned EGCG and ECG downregulate secretion of TNF-α, IL-1β, and IL-6 by lowering p65 expression [79]. Cells treated with glutamine show downregulated iNOS and COX2 expression, as well as NO, PGE2, IL-1β, TNF-α, and IL-8 production [80]. These changes occur via attenuating ERK, p38 and JNK phosphorylation, impairing nuclear translocation of NF-κB and upregulating MAPK-1, which is an inhibitor of ERK kinase [80]. Platelet-rich fibrin extract (PRFe)—a concentrate of platelets with blood constituents—contains a variety of growth factors that may be helpful in regeneration processes [81]. In vitro studies show PRFe mitigates IL-1β IL-6, IL-8, VCAM-1 and ICAM-1 production [82]. It also upregulates odontoblastic proteins—DSPP, dentin matrix acidic phosphoprotein 1 and activates ALP [82].

#### 3.2.2. Fibroblasts

Two studies examined how *P. gingivalis* LPS affects pulp fibroblasts. Only IL-8 (among IL-1b, IL-6, IL-8 and TNF-α measured) was detectable in LPS-stimulated fibroblasts, secreted in a time- and dose-dependent manner [83]. MicroRNA-181 family controls inflammation by regulating growth, development and activation of cells. In LPS-induced fibroblasts, miR-181a expression decreases in a time- and dose-dependent manner, miR-181b expression is barely detectable and miR-181c is absent [83]. LPS enhances chemokine (C-C motif) ligand 3 (CCL3) production in fibroblasts from permanent and deciduous teeth, whereas C-X-C motif chemokine 12 (CXCL12) production is elevated only in fibroblasts from deciduous teeth [84]. CCL3 and CXCL12 take part in leukocytes’ recruitment and activation in acute inflammation.

#### 3.2.3. DPSCs

LPS activates NF-κB signaling pathway in dental pulp stem cells, but its binding activities disappear by 60 min [85].

### 3.3. Other Groups

#### 3.3.1. hDPCs

Nakane et al. [86] postulated that there are differences between *E. coli*’s LPS and LPS produced by *P. gingivalis*, *P. endodontalis* and *F. nucleatum*. The one by *E. coli* inhibits cell protein production more potently than other types. All types of LPS induce DNA production by dental pulp cells [86]. Different types of LPS exacerbate the inflammation, inducing expression of pro-inflammatory cytokines. IL-1β production was enhanced by *P. endodontalis* LPS in cultured human gingival fibroblasts and monocytes from patients with periodontitis in a time- and dose-dependent manner [87] and by *F. nucleatum* LPS in a dose-dependent manner [88]. IL-1ra inhibits IL-1b synthesis induced by *F. nucleatum* LPS [88]. *P. intermedia* LPS induces IL-6 expression (probably mainly transcriptional activation) in human dental pulp fibroblast cultures time- and dose- dependently [89]. Such an IL-6 overexpression is higher than upon *Salmonella* LPS stimulation [89]. CD14 co-stimulates IL-6 expression in dental-pulp fibroblasts [89]. LPS upregulates the expression of both substance P (SP) and SP-receptor in DPCs, indirectly inducing expression of proinflammatory cytokines [90]. *P. intermedia* LPS enhances the production of VEGF in DPCs via an sCD14-dependent pathway [91].

#### 3.3.2. Fibroblasts

*P. intermedia* (ATC 25611) LPS enhances the expression of IL-8 mRNA and its release in human dental pulp fibroblasts with a peak at 12 h [92].

#### 3.3.3. Odontoblasts

*P. intermedia* LPS induces expression of the receptor for advanced glycation end products (RAGE) in odontoblast-like mouse cells [93]. It is a multiligand receptor propagating dysfunction of cells in several inflammatory disorders. LPS also prompts the translocation of high mobility group box 1 (HMGB1) from nucleus to secretory lysosomes, mediated by RAGE and NF-κB activation [93]. HMGB1 stimulation leads to increased expression of RAGE by human microvascular endothelial cells, production of cell adhesion molecules (ICAM and VCAM) and secretion of proinflammatory cytokines: TNFα and IL-8 [93].

*Aggregatibacter actinomycetemcomitans* (ATCC29524) LPS was used in one research on rat clonal dental pulp cells with odontoblastic properties [94]. Ozonated water (O3aq) suppresses calcification and immunologic responses. It also protects against direct damage to the cellular wall [94]. These effects may be achieved by directly inhibiting lipid A by O3aq [94].

#### 3.3.4. DPSCs

*Pseudomonas aeruginosa* LPS has a toxic effect on DPSCs in a dose-dependent manner [95]. Pretreatment with static magnetic field (SMF) attenuates inflammatory response and increases viability of cells. SMF can also enhance the proliferation of DPSCs [95].

### 3.4. Non-Specific

#### 3.4.1. hDPCs

Several studies did not provide LPS sources. As mentioned before, LPS causes aggravation of ROS activity in cells. These particles may damage DNA and activate inflammation. LPS stimulation elevates levels of γ-H2A.X, which is a marker of double strand DNA breaks [96]. This leads to overexpression of transcription factor GATA-4, which stimulates NF-κB pathway by nuclear translocation of p65 with subsequent expression of IL-1β, IL-6 and TNF-α [96]. LPS-stimulated cells show upregulated Lin28 (RNA-binding particle) protein expression, followed by downregulation of let-7b, let-7g and miR98, and upregulation of let-7a, let-7c, let-7d, let-7e, let-7f and let-7i [97]. Let-7 family is a group of microRNAs regulating cell cycle [98].

LPS also stimulates NLRP6 expression and its knock-down decreases IL-1β production [99]. Lipopolysaccharide also upregulates both C5a and C5aR (parts of the complement system) mRNA especially on the second day post-stimulation [100]. Inflammation also raises MMP3 levels. MMP1 production increases upon TNF-α stimulation, but not LPS stimulus [101].

hDPCs possess systems counteracting excess inflammation, such as Angiogenic factor with G patch and FHA domains 1(AGGF1) [102]. It is upregulated in LPS-treated cells and its knock-down activates NF-κB via promotion of phosphorylation of p65 and its transfer to the nucleus [102]. Another similarly functioning compound is GPR173 protein [103]. It is a cognate receptor of a newly discovered hormone, phoenixin-20 [104]. GPR173 is downregulated upon LPS stimulation, leading to induced MMP-2 and MMP-9 expression [103]. However, stimulation with phoenixin-20 reverses this negative effect and decreases expression of TLR4 and MyD88 proteins [103].

Lipopolysaccharides may induce autophagy in hDPCs, e.g., by elevating levels of beclin I and LC3-II and subsequent activation of p38 (MAPK pathway) [105]. Autophagy deactivation is associated with pyroptotic cell death mediated through NLRP3 and Casp1 [106]. Rapamycin—an inductor of autophagy—decreases concentration of pyroptotic mediators—IL-18 and Casp1, subsequently improving LPS-impaired cells viability [106]. An opposite effect is obtained with 3-methyladenine (3-MA), which mitigates autophagy. The key protein regulating autophagy is NF-κB [106].

Another way LPS works is by activating transient receptor potential vanilloid 1 (TRPV1) canal. Overexpression of SIRT6 protein, which promotes degradation of TRPV1 via its ubiquitination, reduces expression of cytokines—IL-6, IL-1β, and TNF-α, deactivates NF-κB and lowers DMP1 level [107]. Capsaicin stimulates TRPV1 in SIRT6-overexpressed cells, counteracting anti-inflammatory properties [107].

Some anti-inflammatory compounds were studied in LPS-treated cells. Eckols—6-6 bieckol (EB1) and pholorofucofuroeckol-A (EB5) are derived from brown seaweed marine algae (Eisenia bicyclis). They downregulate VCAM-1 and ICAM-1 expression via inhibition of ERK1/2 phosphorylation [108]. Moreover, EB1 mitigates COX-2 expression. Eckoles promote ALP activity and expression of osteogenic molecules [108]. Specific miR-146a/PEG-PEI nanoparticles combined with alginate hydrogel with basic fibroblast growth factor (bFGF) stimulate proliferation and promotion of DMP-1 and DSP protein in LPS-stimulated hDPCs [109]. Treatment only with miR146a/PEG-PEI downregulates DMP-1 expression [109]. Saxagliptin (DDP4 inhibitor) reduces oxidative stress, mitochondrial dysfunction, and levels of IL-1β, IL-8 and TNFα [110]. Mineral trioxide aggregate is used in clinical practice as a root repair material. Calcium silicate, compared with MTA, improves cells’ viability, proliferation and reduces IL-1β levels [111]. Both Biodentine and MTA promote expression of DSPP, proliferation and adhesion of cells via AKT pathway [112].

NG2+ cells are a subtype of hDPCs [113]. They are stem cells derived from mesenchymal tissue. Upon LPS stimulation, NG2+ cells produce more IL-1β and IL-6 than hDPCs. NG2+ cells also show enhanced proliferation rate, migration ability and odontoblastic differentiation—changes comparable with the control pulp cells [113].

#### 3.4.2. Fibroblasts

LPS treatment induces labeling of membrane attack complex on the cell surface [114]. It also triggers increased production of C5a—a chemotactic factor, taking part in recruiting inflammatory cells and pulp cells responsible for regeneration [114]. LPS-stimulated fibroblasts have increased mRNA and protein expressions of myocyte-enhancer factor 2 (MEF2C), platelet endothelial cell adhesion molecule-1 (PECAM1) and CXCR4 [115]. PECAM1 expression is positively correlated with B-cell signaling pathways, it also plays a role in angiogenesis [115]. Binding PECAM1 to CXCR4 may intensify inflammation and apoptosis via NF-kB signaling pathway [115]. LPS upregulates NLRP3 inflammasome and pro-IL-1β expression via TLR4/MyD88/NF-κB pathway [116]. miR-223 is involved in the maintenance of the ATP+LPS-induced production and secretion of the proinflammatory cytokines IL-1β and IL-18 mediated by the NLRP3/CASP1 pathway, targeting NLRP3 [116].

#### 3.4.3. Odontoblasts

In odontoblasts, LPS activates the transcription factor FoxO3a 24 h after LPS stimulation [117]. It is accompanied by a rise in autophagy markers, thus protecting cells from death at an early stage of inflammation [117]. The dentin-odontoblast complex may protect cells from apoptosis [118]. LPS increases production of extracellular vesicles (EVs), especially the exosomal components, attenuating apoptosis [118].

TRL4 and NOD2 are expressed in odontoblasts layer more abundantly than in other human pulp stroma cells, which can provide significant defense and anti-infection responses of the dental pulp. After stimulation of preodontoblast mouse cells with LPS, levels of TLR4, NOD2 IL-1β and autophagy proteins (LC3II, beclin1) increased. LPS induced autophagy is associated with TRL4 activation [119].

#### 3.4.4. DPSCs

LPS inhibits the expression of let-7c-5p in rat dental pulp cells [120]. Let-7c-5p may reduce the production of pro-inflammatory cytokines, restoring the viability of cells by suppressing the nuclear translocation of NF-kB p65. A drop in its expression leads to increased expression of dentin matrix protein 1 (DMP1), higher production of pro-inflammatory cytokines and decreased cellular viability [120]. LPS stimulation also regulates other microRNAs: elevates expression of miR-146a-5p, -92b-5p, -218-5p, -23b-5p, -2110, -27a-5p and -200b-3p and decreases in miR-223-3p, -1246 and -494-3p [121]. miR-223-3p may play a role in promoting the angiogenesis of HUVECs [121]. A higher expression of miR-506 and downregulation of Sirtuin 1 (SIRT1) are observed in LPS-induced hDPCs—a finding closely associated with activation of the TLR4-NFkB pathway and expression of pro-inflammatory cytokines: IL-1β, IL-6, and TNF-a [122]. Human β-Defensin 4 (HBD4) exerts anti-inflammatory effects in vitro and promotes the mineralization of DPSC [123].

Inflamed DPSCs do not differentiate properly because of decreased Wnt4 expression [124]. Restoring Wnt4 improves the process, likely via JNK signaling pathway [124] (Table 1).

LPS changes the content of small extracellular vesicles (sEVs), promoting dental pulp regeneration by increasing stem cells migration and elevating the expression levels of repair-associated proteins [125]. Exomes isolated from LPS-induced HDPSCs present a stronger pro—angiogenic effect on human umbilical vein endothelial cells (HUVECs) than HDPSCs without LPS stimulation [121]. A time- and dose-dependent increase in VEGFA expression is also noted [126]. In VEGFA-stimulated hDPSCs, FAK, PI3K, Akt and p38 signaling are activated, possibly enhancing cellular migration [126].

Human platelet lysate (HPL) is a growth factors-rich concentrate of platelets. 20% HPL increases expression of pro-angiogenic factors at both gene and protein levels, while maintaining the cell viability [127]. In LPS-induced DPSCs simvastatin inhibits the expression of proinflammatory cytokines and VEGF, blocking the MAPK signaling [128]. It also promotes proliferation and apoptosis [128].

## 4. Discussion

**Figure 2:**
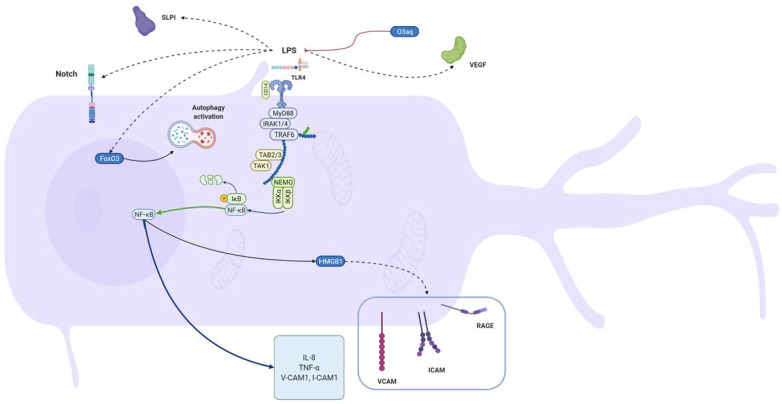
**Molecular effects exerted by lipopolysaccharides on odontoblasts.** Figure summarizes results from studies conducted on hDPCs, regardless of LPS origin. In odontoblasts a main role in LPS stimulation is played by the TLR/MyD88/NF-κB pathway. This stimulation results in expression of IL-8, TNF-α and adhesion molecules. A variety of different pathways and particles activated in LPS-treated cells are presented both in deep blue brackets and by protein images with their names. Black arrows represent positive stimulation, dashed arrows show indirect activation, red ones—inhibition, green—translocation to another cell compartment. Created with BioRender.com 7 December 2020.

**Figure 3:**
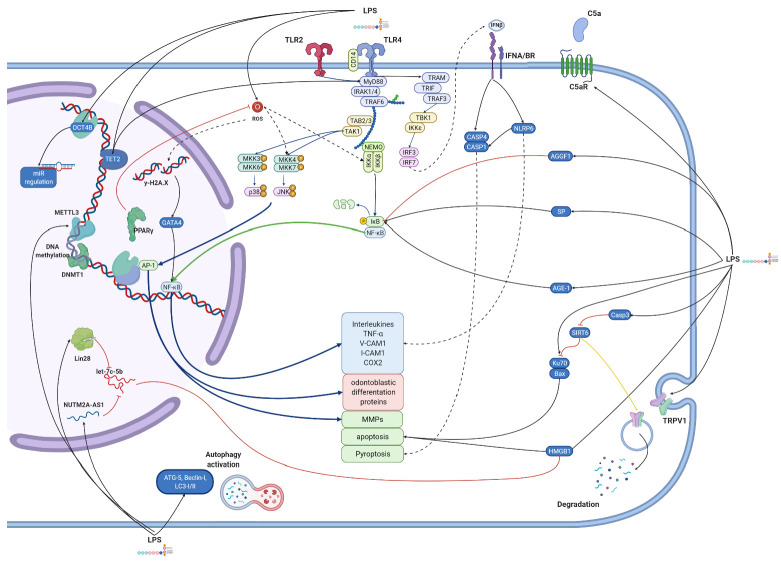
**Molecular effects exerted by lipopolysaccharides on human dental pulp cells (hDPCs).** Figure summarizes results from studies conducted on hDPCs, regardless of LPS origin. The main pathway activated by LPS involves TLR2/4 receptor. Groups of proteins transducing signal are colored differently. TLR pathway, via NF-κB and MAPK, promotes release of interleukins (mainly IL-1β, IL-6 and IL-8), adhesive molecules (I-CAM, V-CAM), metalloproteases (MMPs) and odontoblastic differentiation proteins. This pathway is marked with thick blue arrows. LPS increases ROS activity in cells, changes microRNA profile, leads to apoptosis or pyroptosis. A variety of different pathways and particles activated in LPS-treated cells are presented both in deep blue brackets and by protein images with their names. Black arrows represent positive stimulation, dashed arrows show indirect activation, red ones—inhibition, yellow—ubiquitination, green—translocation to another cell compartment. Created with BioRender.com 7 December 2020.

**Figure 4:**
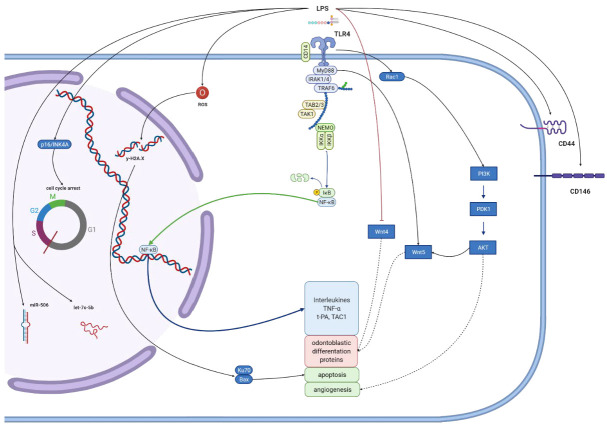
**Molecular effects exerted by lipopolysaccharides on dental pulp stem cells (DPSCs).** Figure summarizes results from studies conducted on DPSCs, regardless of LPS origin. The main pathway is TLR/MyD88/NF-κB. This pathway is marked with thick blue arrows. Its activation increases interleukins production and odontoblastic differentiation. The last one is also mediated by Wnt4/5 pathways. LPS stimulation also causes expression of membranous CD44 and CD146. Variety of different pathways and particles activated in LPS-treated cells are presented in both deep blue brackets and by protein images with their names. Black arrows represent positive stimulation, dashed arrows show indirect activation, red ones—inhibition, green—translocation to another cell compartment. Created with BioRender.com 7 December 2020.

**Figure 5:**
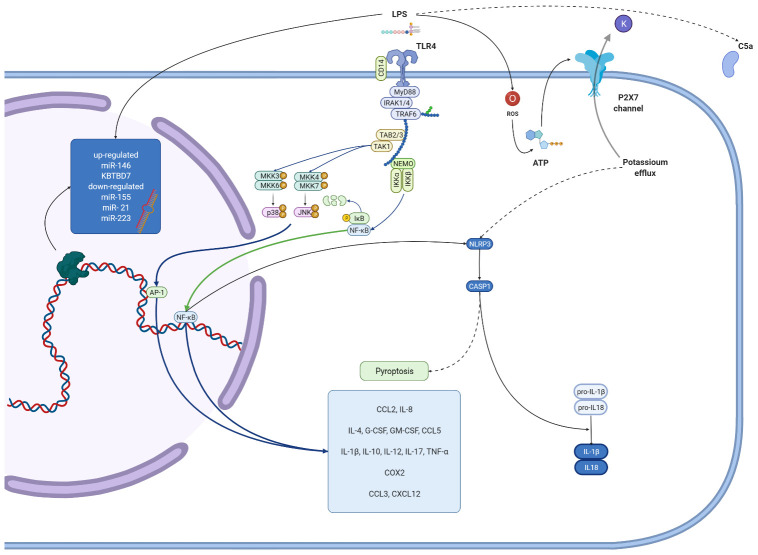
**Molecular effects exerted by lipopolysaccharides on fibroblasts** Figure summarizes results from studies conducted on fibroblasts, regardless of LPS origin. TLR pathway acts via NF-κB and MAPK, promotes release of interleukins, chemokines and growth factors. Deep blue arrows present this pathway. Pathways and particles activated in LPS-treated cells are presented in both deep blue brackets and by protein images with their names. Black arrows represent positive stimulation, dashed arrows show indirect activation, red ones—inhibition, green—translocation to another cell compartment. Created with BioRender.com 7 December 2020.

In this study, we summarize in vitro research concerning LPS influence on different dental pulp cells. Results show that bacterial endotoxins activate a variety of cellular pathways. Figure 2, Figure 3, Figure 4 and Figure 5 give an overview of such mechanisms in particular types of cells. We combined data extracted from analyzed studies with information obtained from KEGG database [129]. Despite some structural differences in LPS originating from miscellaneous bacteria species, they are recognized by only two types of TLR receptors—TLR4 and TLR2, which act via the same pathway. Hence, we simplified the graphical presentation of data, not differentiating between various LPS origins. The main response axis in dental cells begins with activation of TLR4/MyD88/NF-κB pathway, with co-activation of MAP kinases. These pathways are responsible for increasing expression of interleukins, chemokines, MMPs, TNF-α and adhesion molecules. LPS stimulation alters cellular functions in complex ways. Firstly, acting through TLR2/4 receptors, it activates production of inflammatory compounds. Subsequently, gene expression is altered by DNA methylation or expression of microRNAs. LPS also induces autophagy. Finally, LPS stimulation may result in apoptotic or pyroptotic death of affected cells.

An indirect effect of LPS on the inflammation apparatus is exerted by raising ROS production. These factors induce both MAPK and NF-κB pathways, but also inflict DNA damage. This may induce repairing systems or prompt apoptosis, if uncontrolled. Interestingly, agonistic stimulation of PPARγ, which is downregulated in LPS-treated cells, can decrease ROS activity [17].

Apart from TLRs, the NLRP system is also involved in signal transduction after exposure to LPS. In selected studies two NLRPs were described—NLRP3 and NLRP6. They both are involved in transducing the signal for Casp1, resulting in pyroptotic cell death.

One of the well-described pathways in hDPCs and odontoblasts is HMGB1 pathway. In LPS-treated cells, the level of HMGB1 is increased. A potential regulator of this protein is microRNA let-7c-5p, which is downregulated by NUTM2A—a particle expressed abundantly after LPS stimulation. HMGB1 through activation of RAGE receptors deteriorates cellular viability and suppresses proliferation.

The analyzed studies present ambiguous results regarding odontoblastic differentiation. On the one hand, authors show results proving that LPS stimulates cells to become odontoblasts [39,61]—an effect regulated by increased levels of DMP-1, DSPP and ALP activity. An alternative pathway mediating this process may be through activation of Wnt5 or AP-1 and MAPK [40]. On the other hand, however, there are several studies with opposing results. Inhibition of Wnt4 pathway or higher expression of dentine-related proteins occurred in cells treated with compounds reducing expression of proinflammatory interleukins [28,82,124].

The conclusions of the studies presented above should be interpreted with caution, taking into consideration potential biases. Firstly, there is some uncertainty about cells called hDPCs, which were used in most selected studies. As mentioned in the introduction, dental pulp contains a variety of cell types. In most studies, cell isolation methods did not ascertain the purity of isolated cell lineage. Likely, hDPCs are in fact cohorts of non-homogenous cells present in the dental pulp. This poses a risk of bias when comparing study results.

Inflammation is a stressful condition, which changes cellular metabolism. It activates a variety of proteins; therefore, it is also important to consider potential bias associated with crosstalk between different pathways. Most studies analyzed only one particle and potential downstream transcriptional molecules. Such an approach does not consider the potential impact of other molecular mechanisms that may have a reciprocal effect on each other. For example, in studies investigating potential anti-inflammatory compounds, a given molecule may activate some transduction pathways, even though it attenuates the inflammation. To ensure the quality of future studies, proper defining of the phenotype of utilized cells should be a standard practice.

## 5. Conclusions

In summary, LPS affects various proinflammatory pathways in pulp cells. The main role is played by TLR receptor activation followed by NF-κB stimulation. Other important molecules in LPS-stimulated inflammation are NLRs and ROS. Changes in cells treated with LPS are exerted at all levels of expression regulation, from DNA methylation to mRNA post-translational modification.

## Figures and Tables

**Table 1:** Table summarize type of cells, origin of LPS and effects of LPS stimulation of selected studies.

Cells	LPS	Effect in LPS-Stimulated Cells	Author
hDPCs from healthy molar tooth	*E. coli* LPS 0111:B4(L5293)	Higher NLRP6 expression. Knockdown of NLRP6 inhibits IL-1B expression.	[99]
hDPCs from healthy molar tooth	*E. coli* 055:B5	Upregulated HMGB1. Increased DPCs mobility.	[14]
hDPCs from healthy molar tooth	*E. coli* 055:B5	SITR6 promotes Ku70 deacetylation in LPS-stimulated cells; suppresses Bax protein release and apoptosis.	[22]
hDPCs from healthy molar tooth	*E. coli* O111:B4	High level/activation of PPARγ inhibits expression of MMP2, MMP9, VCAM-1 and ICAM-1	[18]
hDPCs and NG2+ from healthy premolar tooth	unspecified	Upregulated IL-1β, IL-6, IL-8, and TNF-α in hDPCs and NG2+. The latter show enhanced proliferation, migration, odontoblastic differentiation.	[113]
hDPCs	*E. coli* 0127:B8	Augmented AGE-1 protein expression. Silencing AGE-1 attenuates expression of IL-1β, IL-6, TNF-α; decreases nuclear translocation of p65 from NF-κB pathway	[19]
hDPCs from healthy molar tooth	*E. coli*	Betamethasone reduces expression of IL-1β, IL-6, TNF-α in LPS induced cells; upregulates ALP, OCN and DSPP proteins	[29]
hDPCs—homogeneous, spindle-shaped fibroblasts	*E. coli*	Elevated TET2 mRNA and protein levels. TET2 knockdown inhibits LPS-induced inflammatory response in hDPCs by downregulating MyD88 hydroxymethylation.	[7]
hDPCs from American Tissue Culture Collection (ATCC, Manassas, VA, USA)	*E. coli* 055:B5	Increased NUTM2A-AS1 levels; lowered expression of let-7c-5p. High level of HMGB-1 attenuates hDPCs vitality, induces apoptosis and expression of IL-6 and IL-8.	[13]
hDPCs from canine, premolar and molar healthy teeth	unspecified	Biodentine and MTA did not decrease level of inflammation related miR-146a but enhanced dentinogenesis, proliferation and adhesion features of cells.	[112]
hDPCs from healthy permanent teeth	*P. gingivalis*	ECG and EGCG reduce mRNA expression and IL-1β, IL-6, TNF-α secretion by interfering with NF-κB pathway and reducing p65 levels.	[79]
hDPCs—dental-pulp fibroblasts	*P. intermedia*	Upregulated mRNA expression for Substance P and its receptor. Substance P enhances LPS-induced expression of IL-1α and COX-2; increases LPS-induced NF-κB binding activity.	[90]
hDPCs—dental-pulp fibroblasts	*P. intermedia*, *Salmonella abortusequi*, *E. coli*	CD14 is costimulatory in IL-6 expression in dental-pulp fibroblasts. Higher IL-6 activity in *P. intermedia* culture than in Salmonella.	[89]
hDPCs from healthy molar tooth	*P. gingivalis*	Increased levels of IFN-β1 (after 3h), NLRP6, CASP1, CASP4 (after 6h). Blocking IFNAR1 lowers expression of those particles. Higher expression of IL-1β.	[73]
hDPCs from healthy molar tooth	*E. coli* 0111:B4	Decreased ATG-5, Beclin-I and LC3-I/II (autophagy), HO-1 and PGC-1α (mitochondria). Schisandrin C treatment recovered expression of all mentioned proteins.	[23]
hDPCs from healthy molar tooth	unspecified	Inhibited GPR173 mRNA and protein expression -maximal effect at 24h. Phoenixin20 inhibits expression of MMP2 and MMP9; suppresses TLR4 and MyD88. The effect of phoenixin20 depends on GPR173.	[103]
hDPCs immortalized by transfection with a human telomerase transcriptase gene (HPD-hTERTs)	*E. coli* 0111:B4	Higher IL-1β, IL-6 expression. Induced Wnt5a, Runx2, and ALP (markers of dentinogenesis). Box-5 (Wnt5a antagonist) treatment mitigates expression of Runx2 and ALP.	[40]
hDPCs immortalized by transfection with a human telomerase transcriptase gene (HPD-hTERTs)	*P. gingivalis*	LPS with TNF-α upregulates VEGF and SIRT1 with subsequent upregulation of MMP-2 and MMP-9. Pathway that leads to this activation involves PI3K, p38, ERK, JNK and NF-κB.	[75]
hDPCs and from healthy premolar tooth	unspecified	Upregulated AGGF1 protein inhibits phosphorylation of NF-κB and its transfer into the nucleus.	[102]
hDPCs from healthy molar tooth	unspecified	Upregulated expression of GATA4, γ-H2A.X and p65. Higher ROS levels cause double-strand breaks. Subsequently, γ-H2A.X upregulates GATA4 and intensifies p65 translocation into nucleus. Elevated IL-1β, IL-6, TNF-α levels.	[96]
hDPCs	unspecified	EB1 and EB5 lower ICAM-1 and VCAM-1 expression, inhibiting ERK and JNK kinases and blocking translocation of NF-κB into nucleus. EB1 mitigates COX-2 expression.	[108]
hDPCs from healthy molar tooth	*E. coli*	Increased production of MMPs.	[6]
hDPCs from healthy molar tooth	*E. coli* 0111:B4	Overexpressed miR-21-5p decreases NF-κB p65 phosphorylation, expression of IL-6, interfering with TRAF6 mRNA, part of TLR4 transduction complex.	[15]
hDPCs from healthy molar tooth	*E. coli*	EGCG and ECG reduce IL-6, IL-8, VCAM-1 and ICAM-1 protein expression.	[36]
hDPCs from healthy tooth	*E. coli*	EGCG and ECG reduce LPS-mediated VEGF production. Catechins attenuated COX-2 expression.	[37]
hDPCs from healthy tooth	*P. gingivalis* AEI2, *P. endodontalis* AE51, and *F. nucleatum*	*E. coli* LPS inhibits cell protein production more potently than LPS from other types. Unlike others, the *E. coli* LPS induces DNA production by dental pulp cells.	[86]
hDPCs from healthy premolar tooth	*P. gingivalis*	Downregulation of mRNA for DNMT3B and protein level of DNMT1. Inhibition of those protein results in upregulation of miR-146a-5p.	[70]
hDPCs from healthy tooth	*P. intermedia*	Enhanced VEGF production	[91]
hDPCs from healthy tooth	*F. nucleatum*	Both LPS and IL-1ra reduce IL-1β synthesis.	[88]
hDPCs from healthy premolar tooth	*E. coli*	Fluocinolone acetonide upregulates DSPP, RUNX2, inhibiting phosphorylated NF-κB p65 and activating AP-1. FA may restore expression of ALP, RUNX2 and DSPP.	[28]
hDPCs from healthy premolar or molar teeth	unspecified	Overexpression of Lin28 RNA binding protein lowers let-7b, let-7g and miR98 levels.	[97]
Primary human dental pulp cells were purchased from American Type Culture Collection (ATCC).	*E. coli* and *P. gingivalis*	Tenegliptin reduces production of 4-HNE from lipid peroxidation as the result of ROS activity in LPS stimulated cells. In addition, suppresses TLR4 mRNA and protein levels after LPS treatment.	[26]
hDPCs from healthy molar tooth	unspecified	Upregulated C5a and C5aR mRNA and protein expression	[100]
hDPCs from healthy premolar tooth	*E. coli* 0111:B4	Upregulated lncMEG3; its knock-down inhibits the secretion of IL-1β, IL-6, TNF-α and promotes odontogenic differentiation through Wnt/β-catenin pathway.	[12]
hDPCs from healthy molar tooth	unspecified	Specific miR-146a/PEG-PEI nanoparticles combined with alginate hydrogel with basic fibroblast growth factor stimulate cell proliferation and expression of DMP-1 and DSP protein.	[109]
hDPCs from healthy molar tooth	*E. coli* 0111:B4	Higher levels of OCT4B1 leading to decreased percentage of apoptotic cells in viability tests.	[9]
hDPCs from healthy molar tooth	*E. coli*	Induced expression of sclerostin; subsequent increase of proinflammatory cytokines through NF-κB pathway. Sclerostin induces expression of VCAM-1 and ICAM-1 and inhibits odontoblastic differentiation. LPS upregulates VEGF, VEGFR and PlGF expression; effect aggravated by sclerostin.	[54]
hDPCs immortalized by transfection with a human telomerase transcriptase gene (HPD-hTERTs)	*E. coli* and *P. gingivalis*	MDP + TLR ligands treatment increases NOD2 and hBD2 mRNA and protein levels compared with MDP + TLR2/TLR4.	[76]
hDPCs from healthy molar tooth?	*E. coli* 0111:B4	Davallialactone eliminates ROS from LPS-induced cells. Decreased expression of ICAM-1, VCAM-1, MMP-2, MMP-9, iNOS and COX-2, caused by inhibiting inflammation at the ERK1/2 and NF-κB stages.	[24]
hDPCs from healthy molar tooth	*E. coli* 0111:B4	Terrein reduces ICAM-1, VCAM-1 levels by blocking Akt-1 action and suppressing NF-κB activation.	[34]
hDPCs from healthy molar tooth	*E. coli*	Upregulated Oct-4B1 and Oct-4B mRNA levels. Oct-4B1 knock-down downregulates Oct-4B and increases number of apoptotic cells. Absence of Oct-4B1 change expression pattern of 38 microRNA.	[8]
hDPCs from healthy molar tooth	*P. gingivalis*	GV1001 peptide inhibits LPS-induced IL-6, TNF-α expression. Effect mediated by reduced phosphorylation of ERK and p38 MAP kinases.	[78]
hDPCs from maxillary supernumerary incisors and molar teeth	*P. gingivalis*	PRFe attenuate IL-1β, IL-6, IL-8, VCAM-1, ICAM-1 expression; enhances dentin sialophosphoprotein, dentin matrix acidic phosphoprotein 1 expression and increases ALP activity	[82]
hDPCs from healthy molar tooth	*E. coli* 0111:B4	Decreased PPARγ levels, ERK1/2 activities and NF-κB translocation. Rosiglitazone decreases proinflammatory stimulation. Activity of PPARγ mediated by removal of ROS formation.	[17]
hDPCs immortalized by transfection with a human telomerase transcriptase gene (HPD-hTERTs)	*P. gingivalis*	Glutamine reduces iNOS and COX-2 expression, inhibits IL-1β, IL-8, TNF-α production and attenuates MAP kinases phosphorylation, NF-κB-p65 nuclear translocation; induces MKP-1 expression.	[80]
hDPCs from healthy deciduous tooth	unspecified	TNF-α and LPS stimulation increases MMP-1 and MMP-3 mRNA levels, but only MMP-3 protein.	[101]
hDPCs from healthy deciduous tooth	unspecified	Simvastatin reduces LPS-stimulated IL-1β, IL-6, VCAM-1, ICAM-1 production, attenuating phosphorylation and translocation of p-65 and I-κB.	[30]
hDPCs from healthy molar tooth	unspecified	Induced expression of beclin-1 and LC3II (autophagy) via MAP kinases phosphorylation and translocation of NF-κB into the nucleus.	[105]
hDPCs from healthy premolar or molar teeth	unspecified	In odontogenic induction medium: increased ALP activity, DSPP-1 and DMP-1 expression. Enhanced NF-κB translocation into nucleus.	[39]
hDPCs from healthy premolar or molar teeth	unspecified	SIRT6 overexpression downregulates IL-1β, IL-6, TNF-α and inhibits NF-κB pathway; reduces expression of TRPV1, whose activation by capsaicin upregulates expression of proinflammatory cytokines.	[107]
hDPCs from healthy permanent teeth	*P. endodontalis*	Increased IL-1β mRNA and protein level in a dose-dependent manner.	[87]
hDPCs from human supernumerary teeth	*E. coli*	Bromelain reduces IL-1β, IL-6, IL-8, VCAM-1 and ICAM-1 expression by inhibiting phosphorylation of p65 protein, ERK and p38 kinases; increases ALP activity.	[33]
hDPCs from human supernumerary teeth	unspecified	Promotion of pyroptotic cell death, with increased levels of IL 1β, IL 18 and caspase 1. Promotion of autophagy inhibits pyroptotic cell death.	[106]
hDPCs from healthy molar tooth	*E. coli* 0111:B4	The antioxidant effect of sole N-acetylcysteine (NAC) not evident. In combination with Biodentine or MTA, improved LPS-induced hDPCs survival at 24 h. NAC+MTA promoted mineralization.	[27]
hDPCs from healthy molar tooth	*P. gingivalis*	Elevated cell apoptosis (higher caspase 3 activity) inhibited cell proliferation and survival, lower SIRT6 expression. SIRT6 has anti-apoptotic effect via regulating Ku70 protein deacetylation.	[74]
hDPCs from healthy premolar and molar teeth	*E. coli*	Upregulated secretion of IL-6 and IL-8. 5-Aza-CdR can promote LPS-induced inflammation by upregulating proinflammatory cytokines expression and activating NF-κB and MAPK signaling pathways by decreasing the methylation level of TRAF6 promoter.	[11]
Primary human dental pulp cells were purchased from American Type Culture Collection (ATCC).	unspecified	Upregulated DPP-4 expression. Saxagliptin ameliorated LPS-induced oxidative stress, mitochondrial dysfunction, apoptosis, and reduced LPS-induced production of TNF-a, IL-1b and IL-8; inhibited p38 activation and NF-κB signaling pathway.	[110]
hDPCs from healthy premolar tooth	unspecified	MTA stimulates IL-1β expression and apoptosis. CS cement enhances cellular proliferation.	[111]
hDPCs from healthy premolar and molar teeth	*E. coli*	Decreased expression of methyltransferase DNMT1, leading to increased cytokine secretion and activating NF-κB and MAPK signaling. Silencing DNMT1 contributes to downregulating methylation levels at the promoters of IL-6 and TRAF6.	[10]
hDPCs from healthy premolar and molar teeth	*E. coli*	Nell-1 may attenuate LPS-induced inflammation acting via p38 and ERK MAPK, but not JNK MAPK signaling pathway.	[31]
hDPCs from healthy premolar and molar teeth	unspecified	Upregulated expression of m6A and METTL3. METTL3 depletion decreased the accumulation of inflammatory cytokines and suppressed the NF-κB and MAPK signaling pathways. METTL3 modulated the alternative splicing of MyD88.	[72]
hDPCs from healthy molar teeth	*E. coli* 0111:B4	HIF1a suppressed IL-6 expression via SOCS3-dependent downregulation of CEBPb.	[21]
hDPCs from healthy molar teeth	*E. coli* 0111:B4	HIF1a promotes pro-inflammatory mediator synthesis via NF-κB signaling—increased levels of IL1b and TNFα. HIF-1α also upregulates phosphorylation of NF-κB p65 and activation of NF-κB signaling. LPS stimulation induced HIF1a expression.	[20]
hDPCs from healthy deciduous tooth	*E. coli*	Acemannan from Aloe vera, induces proliferation, differentiation, growth factor and extracellular matrix components synthesis in LPS stimulated cells.	[38]
Human pulp fibroblasts	unspecified	Induced membrane attack complex labeling on the cell surface. Increased C5a levels in LPS—treated fibroblasts.	[114]
Human pulp fibroblasts	*E. coli* 055B5	Enhanced IL6 and CH3SH production. LPS doesn’t stimulate IL-1β and TNFβ production	[42]
Human pulp fibroblasts	*E. coli*	Upregulated production of CCL2, IL-8 and Il-6. Less pronounced elevation in IL-4, GCFS, GM-CSF and CCL5, followed by IL-1β, IL-10, IL12p70, IL-17A, TNF- α and INF-λ. Most cytokines stimulated during the first 6 h.	[41]
Human pulp fibroblasts	*P. gingivalis* W83	IL-8 detectable after LPS stimulation.	[83]
Human pulp fibroblasts	*E. coli* O111:B4	Induced release of IL-1β, no effect on NLRP3/caspase-1 inflammasome. ATP + LPS stimulate the pyrogenic P2X7 ATP-gated ion channel, activating the NLRP3/caspase-1 pathway and inducing the maturation and release of IL-1β.	[43]
Human pulp fibroblasts	*E. coli*	COX-2, but not COX-1 was induced by *E. coli* LPS after stimulation for 3 h. Exogenous melatonin suppresses COX-2 and IL-1β.	[45]
Human pulp fibroblasts	unspecified	Increased mRNA and protein expression of MEF2C, PECAM1 and CXCR4.	[115]
Human pulp fibroblasts	*P. intermedia* ATCC 25611	*P. intermedia* LPS expressed IL-8 mRNA and released IL-8 in human dental pulp fibroblast cultures, expression peaked after 12 h.	[92]
Human pulp fibroblasts	*E. coli*	Increased expression of miR-146a in dental pulp cells (also in gingiva and periodontal ligament fibroblasts). Decreased expression of miR-155 in gingival fibroblasts.	[46]
Human pulp fibroblasts from permanent and deciduous teeth	*P. gingivalis*	Increased CCL3 production in cells from both permanent and deciduous teeth. Pulp fibroblasts from deciduous teeth had elevated production of CXCL12.	[48]
Human pulp fibroblasts	*E. coli* 055:B5, L2880	Downregulated miR-21 and upregulated KBTBD7. Berberine reduced expressions of IL-1β, IL-6 and TNF-α, as well as enhanced cell proliferation and miR-21 expression.	[47]
Human pulp fibroblasts	unspecified	ATP + LPS reduced miR-223 expression in human dental pulp cells. miR-223 negatively regulated production and secretion of IL-1β and IL-18, acting via the NLRP3/CASP1 inflammasome pathway by targeting NLRP3.	[116]
Human pulp fibroblasts from premolars	*E. coli* 0111:B4	LPS + ATP increases IL-1β secretion via NLRP3 inflammasome activating caspase-1. The TLR4/MyD88/NF-κB mediates up-regulation of NLRP3 and pro-IL-1β genes expression. ATP promotes ROS production which serves as the second signal for the activation of NLRP3 inflammasome.	[44]
hDPSCs from healthy molar tooth	*E. coli*	DPSCs/NF-PLLA scaffold constructs with simvastatin reversed LPS induced TNF-α, IL-1β and MMP-9 mRNA expression on day 28.	[69]
Adult human dental pulp stem cells	unspecified	20% HPL stimulates angiogenesis by increasing levels of pro-angiogenic factors’ mRNA and protein	[127]
Adult human dental pulp stem cells from first premolars	*E. coli*	Induced expression of IL-6, IL-8, tPA and TAC1 levels. Levels of IL-1α were increasing proportionally to LPS concentrations. TNFα showed increased expression with no changes at gene expression level. This could be attributed to initiation of inflammation via TLR4 and TLR2. The viability was reduced in LPS treated cells.	[55]
human dental pulp stem cells	*P. gingivalis*, *P. endodontalis*	LPS and TNF activated the NF-κB signaling pathway. Stimulation by the latter lasted longer. TNF induced the phosphorylation and degradation of IκBα more potently than LPS. LPS did not induce phosphorylation of p65 transactivation domain, while TNF only weakly stimulated p65 phosphorylation.	[85]
human dental pulp stem cells from third mandibular molars	*E. coli* 0111:B4L2880	4-MU may facilitate cell differentiation by downregulating the expression of inflammatory cytokines. CD44 expression was downregulated upon 4-MU treatment or with LPS. CD44 plays a primary role in HA-induced cytokine release via the formation of the LMW HA-TLR4-CD44 complex. 4-MU could accelerate the LPS-induced migration of hDPSCs.	[62]
human dental pulp stem cells	unspecified	Altered content of dental pulp cells-derived small extracellular vesicles (sEVs). sEVs carry biologically active molecules from parental cells, which can mediate intercellular communication, induce MSC differentiation, and ultimately promote the healing.	[125]
human dental pulp stem cells from third molars	*E. coli* 0111:B4	More pronounced SA-b-gal-positive signal (a maker of senescence), likely resulting from TLR4/MyD88-NF-jB-p53/p21 signaling pathway activation. Knockdown of p65 reversed the senescence of enhanced proliferation and the increased the total number of DPSCs with more organized F-actin.	[65]
human dental pulp stem cells from third molars	*E. coli* 0111:B4	LPS binding with TLR4 generates ROS. The DDR and p16INK4A pathways might be the main mediators of DPSC LPS-induced senescence. ROS production may promote DDR and p16INK4A expression and then cell cycle arrest.	[64]
human dental pulp stem cells from premolars or third molars	*E. coli* 055:B5	SDF-1a secretion largely suppressed, increased production of CXCR4.	[63]
human dental pulp stem cells from third molars	Ultrapure *E. coli*	LPS can enhance Wnt5a expression preincubation with TLR4 neutralizing antibodies reduced LPS-induction Wnt5a expression (mainly activated through the MyD88-dependent pathway). NF-kB activation and PI3K/AKT signal pathways regulate Wnt5a expression.	[58]
human dental pulp stem cells	Ultrapure *E. coli*	Induced odontogenic differentiation by increasing ALP, OCN, DMP1 and DSPP expression and mineralized nodules formation. TLR4 expression maximal after 7 days. Inhibiting or blocking TLR4 decreased the LPS-mediated expression of mineralized tissue markers and nodule formation. NF-kB signaling activated by LPS in a time-dependent manner, but not involved in cellular differentiation. Activation of p38 and ERK MAPK, not JNK MAPK signaling pathways contribute to LPS-induced differentiation of hDPSCs.	[61]
human dental pulp stem cells from wisdom teeth/premolars	*P. aeruginosa*	Dose-dependent toxic effect. (SMF) stimulation inhibits inflammatory response, can enhance proliferation.	[95]
human dental pulp stem cells from third molars	unspecified	Exosomes from LPS-stimulated hDPSCs exert a stronger pro-angiogenic effect on HUVECs than normal hDPSCs—LPS-dose dependently. Expression of 7 microRNAs were increased (miR-146a-5p, miR-92b-5p, miR-218-5p, miR-23b-5p, miR-2110, miR-27a-5p, and miR-200b-3p) and 3 decreased (miR-223-3p, miR-1246 and miR-494-3p). Five of them play important roles in inflammation and HUVEC function and angiogenesis (miR-223-3p being the strongest candidate).	[121]
human dental pulp stem cells from premolars	*E. coli* 0111:B4	High expression of γ-H2A.X. marker of DSB. The mRNA and protein expression levels of Ku70 and Xrcc4 involved in NHEJ, and Rad51 in HR, significantly increased in DPSCs. Ku70 knockdown reduces the expression of XRCC4 and promotes apoptosis of DPSCs during inflammation, thereby Ku70 serves as a link between DNA damage and apoptosis.	[66]
human dental pulp stem cells from third molars	*E. coli*	Upregulated CD146 expression levels. Partially blocked expression of the NF-κB subunit p65 leading to reduced TNF-α production by macrophages. The innate immune response dependent on the TNF-α/IDO axis.	[57]
human dental pulp stem cells from third molars	*E. coli*	EGCG exerted an anti-inflammatory effect on hDPSCs without affecting cell proliferation or differentiation. EGCG inhibits hypoxia-induced apoptosis.	[67]
rat dental pulp stem cells	*E. coli* 055:B5	LPS at low concentrations upregulated mRNA expression of mineralization-related genes (OCN, DSPP, ALP and BSP) in JDPSCs and ADPSCs, LPS effects declined with age. Enhanced proliferation by increasing TLR4 expression and through PI3K/Akt signaling.	[59]
human dental pulp stem cells from third molars	unspecified	VEGFA promoted the migration of hDPSCs in a concentration-dependent manner. VEGFA/VEGFR2 axis interacted with the FAK/PI3K/Akt and p38 MAPK signaling pathways in mediating hDPSCs migration.	[126]
human dental pulp stem cells from premolars (DPSCs) and stem cells from human exfoliated deciduous teeth (SHED)	*E. coli*	Betamethasone blocks NF-κB activation and exhibits an osteo-/odonto-inductive effect on DPSCs and SHED. It also displays an osteoclast effect on SHED, not on the DPSCs.	[68]
rat dental pulp stem cells	unspecified	The expression of miR-506 was high while that of SIRT1 was low, which was associated with pro-inflammatory cytokines upregulation and activation of the TLR4-NFkB pathway.	[122]
human dental pulp stem cells from third molars	*E. coli* O55:B5	Decreased cell survival and more frequent necrosis. Reduced COL1A1 expression after 21 d. Promoted production of IL-6 in the late phase.	[56]
human dental pulp stem cells from third molars	unspecified	Simvastatin promoted cell proliferation, cell cycling and apoptosis in LPS-induced DPSCs. Expression of cytokines and VEGF (via MAPK signaling blockade) was inhibited.	[128]
rat dental pulp stem cells	unspecified	Inhibited expression of let-7c-5p both in vivo and in vitro. The overexpression of let-7c-5p suppressed the production of pro-inflammatory cytokines, restoring viability; also inhibited the LPS-induced activation of NF-kB signaling by inhibiting the phosphorylation of IκBa and IKKb and increasing total IκBa expression, hence suppressing the nuclear translocation of NF-kB p65. let-7c-5p action depends on the inhibition of DMP1 function.	[120]
rat dental pulp stem cells	*E. coli* 055:B5	Induced expression of let-7c-5p could suppress inflammation and restored the osteogenic differentiation potential of inflamed DPSCs. The effect depended on the repression on HMGA2 function by let-7c-5p, leading to inhibiting PI3K/Akt pathway.	[60]
human dental pulp stem cells	unspecified	Human β-Defensin 4 (HBD4) shows anti-inflammatory activity in vitro, by reduction of IL-1α, IL-1β, IL-6 and TNF-α expression and promotes mineralizing cell phenotype differentiation in DPSC. Similar effects are noted in vivo.	[123]
human dental pulp stem cells—normal pulp derived from the mandibular third molar and inflamed pulps derived from pulps of patients with irreversible pulpitis	unspecified	Decreased Wnt4 expression, impairing the odontogenic differentiation of DPSCs. Restoration of Wnt4 was able to rehabilitate the impaired odontogenic differentiation potential. Wnt4 may function through its effect on JNK signaling pathways.	[124]
odontoblast-like cells (MDPC-23), undifferentiated dental pulp cells (OD-21), macrophages, and gingival fibroblasts and human embryonic kidney cells (293T, ATCC)	*E. coli*	Upregulated VEGF expression (TLR4-dependent signaling pathway). Therapeutic blockade of the LPS-TLR4-VEGF pathway might be beneficial for the treatment of teeth with reversible pulpitis.	[49]
odontoblast-like cells (MDPC-23)	*E. coli* k-235 strain	Odontoblast-like cells produce secretory leukocyte protease inhibitor (SLPI) in response to LPS, inhibiting the activation of NF-kB.	[51]
Human odontoblasts from third molars	*E. coli* 055:B5	Upregulated IL-8 mRNA and protein levels.	[50]
self-established pre-odontoblastic cell line from third molars	unspecified	During the early stage of inflammation, FoxO3a might regulate autophagy activation for odontoblast survival.	[117]
Mouse odontoblast-like cells	*E. coli* L-2880	Notch signaling activation by LPS stimulation is similar to that caused by mechanical injury in vivo.	[52]
rat clonal dental pulp cell line with odontoblastic properties (KN-3)	*A. actinomycetemcomitans* ATCC29524	O3aq directly suppresses the biological effects of LPS on calcification and immunologic responses of odontoblast-like cells and its ability to demolish cell walls and cytoplasmic membranes. O3aq effects may be achieved through the direct inhibition of lipid A.	[94]
Human Dental Pulp Tissues; Odontoblast-like cells, OLC-1, obtained from mouse tooth germs	*P. intermedia*	Upregulated danger signals HMGB1 and RAGE. In response to HMGB1 stimulation, human microvascular endothelial cells increase expression of RAGE, cell adhesion molecules, e.g., ICAM-1 and VCAM-1, and the secretion of TNFα and IL-8. The increase of HMGB1 in the cells was blocked by an inhibitor of IKK-β (TPCA-1). *P. intermedia* LPS-mediated HMGB1 translocation involves NF-κB activation in OLC-1.	[93]
Odontoblast-like cells from human dental stem cells from the apical papilla (SLMhSCAP) were primarily cultured from extracted third molar dental papilla	unspecified	Increased exosome production in odontoblast-like cells generated from mineralization medium-treated hSCAPs. The exosomes showed anti-apoptosis functions. These exosome-dependent intercellular pathways may protect cells from LPS-induced apoptosis.	[118]
Mouse odontoblast-like cells	*E. coli*	Propolis decreased production of IL-1α, MIP-1a, IL-12(p70), and IL-15 in odontoblast-like cells.	[53]
Preodontoblastic cell line	unspecified	Levels of TLR4, NOD2 IL-1β and autophagy proteins (LC3II, beclin1) increased.	[119]

Abbreviations: LPS—lipopolysaccharide, IL—interleukins, hDPCs—human dental pulp cells, DPSCs—dental pulp stem cells, NOD/NLRP—nucleotide-binding oligomerization domain-like receptors, NF-κB—nuclear factor kappa-light-chain-enhancer of activated B cells, HMGB1—high mobility group box 1, RAGE—receptor for advanced glycation end products, PPARγ—peroxisome proliferator-activated receptors γ, MMPs—matrix metalloproteinases, VCAM—vascular cell adhesion protein, ICAM—intercellular adhesion molecules, TNF-α—tumor necrosis factor alpha, AGE-1—, ALP—alkaline phosphatase, OCN—osteocalcin, DSPP—dentin sialophosphoprotein, TET2—tet methylcytosine dioxygenase 2, ECG—epicatechin gallate, EGCG—epigallocatechin-3-gallate, COX-2—cyclooxygenase-2, IFN-1β—interferon-1β, ATG-5—autophagy related 5, LC3-I/II—microtubule-associated proteins 1A/1B light chain 3B, HO-1—heme oxygenase 1, PGC-1—peroxisome proliferator-activated receptor gamma coactivator 1-alpha, VEGF—vascular endothelial growth factor, PI3K—phosphoinositide 3-kinases, ERK—extracellular signal-regulated kinases, JNK—c-Jun N-terminal kinases, AGGF1—Angiogenic factor with G patch and FHA domains 1, γ-H2A.X—phosphorylated H2A histone family member X, EB1/5—6-6 bieckol (EB1) and pholorofucofuroeckol-A(EB5), TRAF6—TNF receptor associated factor, AP-1—Activator protein 1, RUNX-2—runt-related transcription factor 2, MEG3—maternally expressed 3, iNOS—inducible Nitric oxide synthases, OCT4—octamer-binding transcription factor 4, PRFe—platelet-rich fibrin extract, MKP-1—MAPK phosphatase 1 (MKP-1),TRPV1—transient receptor potential cation channel subfamily V member 1, NAC—N-acetylcysteine, 5-Aza-CdR—5-Aza-2′-deoxycytidine, DPP-4—Dipeptidyl peptidase-4, MTA—mineral trioxide aggregate, DNMT1—DNA (cytosine-5)-methyltransferase 1, m6A—N6-Methyladenosine, METTL3—N6-adenosine-methyltransferase 70 kDa subunit, HIF1α—hypoxia-inducible factor-1α, SOCS3—suppressor of cytokine signaling 3, CEBPb—CCAAT enhancer-binding protein beta, CH3SH—methyl mercaptan, CCL—chemokine ligand, CXCL chemokine (C-X-C motif) ligand, CXCR—chemokine (C-X-C motif) receptor, G-CSF—granulocyte colony-stimulating factor, GM-CSF—granulocyte-macrophage colony-stimulating factor, P2X7—P2X purinoceptor 7, ATP—adenosine triphosphate, sEVs—small extracellular vesicles, DMP-1—dentin matrix acidic phosphoprotein 1, DSB—double strand break, XRCC4—X-ray repair cross-complementing protein 4, NHEJ—non-homologous end joining, HR—homology directed repair, HBD-4—human beta-defensin 4, SLPI—secretory leukocyte protease inhibitor.

## Supplementary Materials

The following are available online at https://www.mdpi.com/article/10.3390/biom12010138/s1, File S1: Systematic review protocol.
