# Supplementary material for: Role of Lipopolysaccharide, Derived from Various Bacterial Species, in Pulpitis—A Systematic Review
Source: Biomolecules. 2022 Jan 15;12(1):138. doi: 10.3390/biom12010138 (PMC8774278; doi:10.3390/biom12010138)
Supplement: Supplementary file 1 [file biomolecules-12-00138-s001.zip › biomolecules-1521648-supplementary.pdf]

Systematic review protocol

PICO

- Population – dental pulp cells (in vivo experiments excluded)
- Intervention – **LPS (Lipopolysaccharide)** stimulation cells in vitro
- Comparison – control group without LPS stimulation
- Outcome – any (i.e. changes in mRNA or protein expression, viability, mobility etc.)
- Study design – all except for reviews, letters and editorials, English language only

Draft title: Role of lipopolysaccharide, derived from various bacterial species, in pulpitis – a systematic review

SUMMARY

Context: Data regarding the LPS stimulation on dental pulp cells has not been systematically reviewed and analyzed.
Objective: To gather all available evidence on the impact of LPS on dental pulp cells.
Data sources: English articles from MEDLINE, Web of Science and Scopus
Study selection: vide Study design in PICO
Data extraction: Data extraction from articles by MC, followed by a review by M. Kopka, using predefined data fields.
Data synthesis: non-applicable
Conclusions: Collected data give comprehensive summary on LPS effects on different cells present in pulp tissue.

Search engines

Pubmed
(((„Tooth pulp*"[tw] OR "tooth pulp*" OR "dental pulp*") AND ("inflam*" OR inflammation[mh])) OR pulpitis[mh] OR "pulpitis") AND (Lipopolysaccharides[mh] OR "LPS" OR "LPSs" OR "Lipopolysacchari*" OR Bacterial Polysaccharides[mh])

Web of science
((("tooth pulp*" OR "dental pulp*") AND ("inflam*")) OR "pulpitis") AND ("Lipopolysaccharides*" OR "LPS" OR "LPSs" OR "Bacterial Polysaccharides*")

Scopus
TITLE-ABS-KEY ((("tooth pulp*" OR "dental pulp*") AND ("inflam*")) OR "pulpitis") AND ("Lipopolysaccharides*" OR "LPS" OR "LPSs" OR "Bacterial Polysaccharides*") AND (LIMIT-TO (PUBSTAGE , "final")) AND (LIMIT-TO (DOCTYPE , "ar") OR LIMIT-TO (DOCTYPE , "re")) AND (LIMIT-TO (LANGUAGE , "English"))

Search strategy and verification

Step 1. Duplicates removal

Step 2. Screening by title:
- Is LPS used in the study?
- Are pulp cells used in the study?
- Is the subject of this study the use of LPS on pulp cells and investigation of effects?

If 3 x “yes” → Study included in step 3
If at least 1 “no” → Study excluded
If not certain → Abstract screening

Step 3.

- Verification of inclusion and exclusion criteria via full text screening

Inclusion criteria:

- Appropriate PICO
- Reported outcomes

Exclusion criteria:

- Inadequate PICO
- In vivo studies
